# Unfavorable factors in accessing the pelvicalyceal system during retrograde flexible ureteroscopy (fURS)

**DOI:** 10.25122/jml-2023-0005

**Published:** 2023-03

**Authors:** Bogdan Geavlete, Cristian Mareș, Răzvan-Ionuț Popescu, Răzvan Mulțescu, Cosmin Ene, Petrișor Geavlete

**Affiliations:** 1 Sanador Hospital, Bucharest, Romania; 2 Department of Urology, Emergency Clinical Hospital Sf. Ioan, Bucharest, Romania

**Keywords:** flexible ureteroscopy, renal malformations, horseshoe kidney, ectopic kidney, passive deflection

## Abstract

Flexible ureteroscopy (fURS) is a well-established procedure for treating multiple upper-urinary tract pathologies, particularly renoureteral lithiasis. Endoscopes have undergone significant advancements, including miniaturization, improved optics, and increased maneuverability. In addition, advancements in accessory instruments, such as the performance of laser fibers, guidewires, and extraction probes, have played a significant role in improving the overall performance of flexible ureteroscopy procedures. However, despite these advancements, unique circumstances can make achieving optimum results during flexible ureteroscopy challenging. These include congenital renal anomalies (horseshoe kidneys, ectopic kidneys, rotation anomalies), as well as the unique intrarenal anatomy (infundibulopelvic angle, infundibular length) or the specifications of the endoscope in terms of maneuverability (active and passive deflection). This review explored challenging scenarios during flexible ureteroscopy procedures in the pyelocaliceal system.

## INTRODUCTION

In the past three decades, flexible ureteroscopy (fURS) has undergone substantial technological and technical developments, leading to its widespread use for treating various upper urinary tract disorders, primarily urolithiasis. Since Marshall's initial description of fURS in 1964, flexible ureteroscopes have undergone major technological advances [1]. As a result, these devices now exhibit a high success rate in clinical settings, a low incidence of related morbidity, and are relatively easy to use. Marshall's original description of the ureteroscope only allowed passive deflection and lacked a working channel. Later, Takayasu introduced a ureteroscope that integrated active deflection. In 1987, Demetrius Bagley introduced the flexible ureteroscopy as we know it today [2-4].

The successful development of the flexible intracorporeal lithotripter Holmium: Yttrium Aluminium Garnet (Ho: YAG) laser with a good safety margin has raised interest in treating urolithiasis as a retrograde intrarenal surgery (RIRS) [5,6]. More recently, the introduction of the Thulium laser (TFL) marked a new milestone in the evolution of intracorporeal lithotripsy and ureteroscopy, adding value to the already established techniques in this surgical field. It permits the energy to travel more effectively than Ho: YAG in a more focused beam and at a wavelength with a higher water absorption coefficient [7,8]. Studies conducted in vitro and ex vivo with TFL have revealed that less time and energy are needed to remove kidney stones because of their technical characteristics [9,10]. In conjunction with the advancements in laser technology, the anatomy of modern ureteroscopes has also undergone significant changes. Other technological developments in recent years have improved active deflection and reduced scope caliber, resulting in better surgical outcomes and shorter operating times [11]. The transition from optical systems using optical fiber to digital ureteroscopes represented a new achievement in developing modern and durable devices with improved visualization and a longer lifespan [12,13]. The development of ideal smaller diameter endoscopes, enhanced image quality, and maneuverability along with long-lasting durability have been the goals of ongoing technological advancements [14]. The possible benefits of endoscope miniaturization include potential reductions in pre-stenting rates, better irrigation outflow, improved irrigation turnover, better manipulation, and perhaps reduced risk of ureteral injury [15]. The introduction of single-use flexible ureteroscopes has significantly increased their accessibility in the healthcare setting, leading to a rise in the number of minimally invasive procedures for kidney stone removal [16]. Boston Scientific unveiled the LithoVueTM, the first digital single-use ureteroscope, in January 2016 [17]. With innovative single-use technologies, flexible ureteroscopy, and retrograde intrarenal surgery entered a new age (RIRS). The introduction of Pusen's (PusenTM - Zhuhai Pusen Medical Technology Co, Ltd., Zhuhai, China) new 7.5 Fr single-use flexible ureteroscope has revolutionized the flexible ureteroscope industry, introducing a powerful and practical instrument that has the advantage of increased maneuverability, excellent visualization, lightweight, and at the same time does not require resterilization, maintenance costs and does not predispose to cross infections. The thinnest single fURS has an outer shaft diameter of 7.5Fr and a working channel diameter of 3.6Fr. This approach can address accessibility issues without needing an access sheath or a smaller access sheath, thereby reducing procedure-related morbidity and allowing passage through a narrow ureter. This can also be particularly useful in solving more complex cases where access with a standard ureteroscope is challenging [18,19].

However, despite these technical advancements, there are still instances where procedures may not be straightforward. Factors such as anatomical anomalies of the reno-ureteral system, difficult positioning of the calculus at the lower calyx level, or suboptimal maneuverability of the ureteroscope when accessing a certain point in the pelvicalyceal system can make the technique challenging, leading to suboptimal outcomes. This review aimed to evaluate situations where access of the flexible ureteroscope is difficult due to patient-related factors or technical characteristics of the endoscope.

### Renal malformations leading to access difficulties

Patients with renal anatomical anomalies represent a unique population that requires special consideration in managing upper urinary tract lithiasis [20]. Pelvic anatomic anomalies, such as aberrant vessels that obstruct the pelvic ureteric junction (PUJ), PUJ stenosis, and the presence of diverticula or other congenital renal malformations, pose challenges for the urologist when performing flexible ureteroscopy. Different defects in embryological development result in defective kidneys. These could be connected to irregular rotation, ascent, fusion, or a combination of these changes. Horseshoe kidneys (HSK) are the most common congenital renal malformations, with an incidence rate of 1 in 400, while ectopic kidneys (EK) are less commonly observed, with an incidence rate of 1 in 3000 [21]. These structural abnormalities enhance the incidence of urolithiasis and compromise renal drainage [22]. Endourological care is difficult because access to the upper urinary tract is challenging. Although shockwave lithotripsy (SWL) and percutaneous nephrolithotomy (PCNL) can be an option in these cases, the complication rates are higher, and the failure of definitive treatment of the stone is lower compared to anatomically normal kidneys [23-25].

Technological and procedural advancements have greatly expanded the indications for flexible ureteroscopy (fURS), making it a highly effective therapeutic option for complex intrarenal anatomy considering the advent of smaller caliber ureteroscopes enhanced with greater deflection capabilities and modern fiber lasers [26]. A study by Jie Ding [27] investigated the outcomes of fURS in patients with kidney stones and horseshoe kidneys. The study found that the average calculus size was 29±8 mm, with a total operative time of 92±16 minutes. Of the total number of patients, 62.5% achieved stone-free status after the first procedure, and for the remaining patients, the total stone-free rate reached 87.5% after the second procedure. The study reported no major complications associated with the procedure.

Another recent study compared retrograde endoscopic treatment (fURS-RIRS) and percutaneous nephrolithotomy (PCNL) in treating renal lithiasis associated with horseshoe kidneys. The study followed 49 patients who received both treatments, with 21 undergoing PCNL and 28 RIRS [28]. Although the stone-free rates were comparable for both methods, the postoperative complications and perioperative morbidity associated with PCNL were much lower compared to fURS, considering it a viable option for these patients. A recent paper published in late 2021 [29] compared retrograde stone treatment with single-use flexible ureteroscopes (su-fURS) (14 patients) and reusable flexible ureteroscopes (re-fURS) (15 patients) following 29 patients with horseshoe kidneys over 5 years. The study found that both methods resulted in similar outcomes in terms of complications, operation time, mean stone burden, and stone-free rates, concluding that fURS is a safe alternative for treatment in these problematic cases. The study used the PU3022 ureteroscope from Zhuhai Pusen Medical Technology, which demonstrated exceptional accessibility, maneuverability, and operating time, reaffirming the safety and reliability of modern single-use instruments that are economically efficient and do not require maintenance costs. These instruments offer the added advantage of surgeon safety, enabling them to solve particularly difficult cases without fear of damaging expensive, reusable instruments. The authors highlighted the clear advantage of these new instruments, especially in cases where intrarenal access is difficult.

Ectopic kidneys result from impaired embryological development, and patients with this condition are more likely to experience various disorders, including hydronephrosis and nephrolithiasis, due to the abnormal position, orientation, and form of the pelvic kidney. The anatomical and architectural abnormalities associated with ectopic kidneys make it difficult for urologists to treat pelvic kidney stones [30,31]. Many studies have followed the incidence of renal lithiasis in this type of malformation and optimal methods of minimally invasive treatment.

Although ureteroscopy may be difficult in these particular patients, a systematic review conducted in 2020 by Lisa Lavan et al. [32] examined 117 cases of ectopic kidney along with other kidney anomalies and demonstrated that endourological technique advancements had made ureteroscopy an effective and safe procedure, combined with minimal rates of complications and promising postoperative stone-free status. A study conducted by Omer Faruk Bozkurt et al. [33] investigated retrograde fURS management in 26 patients with renal lithiasis associated with ectopic kidneys [33]. The study reported a stone-free rate of 84.6% (22 patients), while 4 patients (15.4%) failed retrograde treatment due to fragment obstruction or the difficult position of the stone in the lower calyx, despite the best possible ureteroscope deflection. Another article published in mid-summer 2021 [34] compared the effectiveness of fURS in managing ectopic pelvic kidneys in 11 patients over 3 years, analyzing mean operative time, hospital stay, stone-free rate, and complications. The stone-free status (fragments <3 mm) after one session was 60.1%, followed by 84.1% after the second session and 94.4% after the third intervention, respectively. The average stone burden was 30 ± 9 mm (17 to 49 mm). In terms of complications, the overall rate was 19.7%, according to the Clavien-Dindo system. The authors concluded that fURS was an efficient treatment strategy with a high stone-free rate and low complication profile in ectopic kidney calculi.

In a comprehensive review published in 2017 in the World Journal of Urology [35], Mahesh Desai compared the current standard treatment options for renal stones, including SWL, fURS, and PCNL, citing guidelines from the American Urology Association and European Association of Urology [35]. It highlighted the important role of each specified procedure in a different situation and concluded that none of the aforementioned techniques is optimal for all situations. Each procedure has advantages and disadvantages, and various cases should be considered individually to achieve the best results. It highlights the importance of fURS in abnormal kidneys when the stone is located in the lower pole measuring 1.5-2cm (considering the unfavorable factors for SWL) or stones located in a diverticulum or a diverticular neck.

The 2022 guidelines of the European Association of Urology on Urolithiasis [36] underline the importance of considering special problems in stone removal, such as calyceal diverticulum stones, horseshoe kidneys, or stones in the pelvic kidney, where a retrograde approach is a feasible option with minimal complications and substantially stone-free rates. A special category of abnormalities is represented by patients with obstruction of the UPJ, where PCNL should be considered the first option in stone removal. However, even in these cases, the retrograde approach can achieve good results if associated with Ho: YAG laser endopyelotomy. When none of the aforementioned procedures succeeds, open surgery to correct the UPJ obstruction (pyeloplasty) and remove stones is the ultimate option. In fact, UPJ obstruction represents perhaps the most important anatomical modification of the kidney that makes it difficult for the ureteroscope to access the pyelocaliceal system. Multiple studies [37-39] suggest the particular complexity of these cases associated with renal lithiasis, which determines other procedures as primary options in treating these patients. A comprehensive review published by Andreas Skolarikos in Urolithiasis [40] aimed to determine the optimal approach of renal stones associated with UPJ obstruction. The review concluded that in these particular cases, the best approach would be laparoscopic or robotic pyeloplasty associated with stone removal in the same operative setting. It is safe to conduct and has a high success rate for maintaining UPJ patency and stone-free status. When treating concurrent renal stones and ureteropelvic junction stenosis, minimally invasive pyeloplasty should be the first option considering that endopyelotomy has poorer long-term obstruction-free rates.

### Difficulties in intrarenal handling of the ureteroscope

With improvements in optical systems, digital video capability, laser lithotripsy, smaller ureteral stone baskets, and dual working channels that enable continuous pressurized irrigation for improved visualization, ureteroscopy has attained the imaging capability, precision, versatility, safety, and reliability needed to become a standard tool in the armamentarium of every urologist. However, with all these improvements, the literature still highlights multiple situations that make it difficult for the ureteroscope to access all the intrarenal areas. One of the most serious situations that complicate ureteroscopy in the retrograde treatment of renal lithiasis is the lower pole stone. Multiple systematic publications have tried to determine objectively the factors that make it difficult to access the lower renal pole [41,42]. Traditionally, it is considered that an infundibulopelvic angle (IPA) <30° and a long infundibular length (IL) (>3 cm) are determining factors [43]. A recent large-scale study published in the International Journal of Urology in October 2022 aimed to determine the specific parameters of the "inaccessible" anatomy of the lower pole in 854 patients with kidney or ureteral stones [44]. The mean values determined were IPA = 54.6°, infundibular width (IW) = 9.4 mm, and calyceal pelvic height (CPH) = 30.9 mm. IPA 45.8° and IW 7.8 mm were unfavorable predictors for accessing the kidney lower pole in fURS.

**Figure 1:**
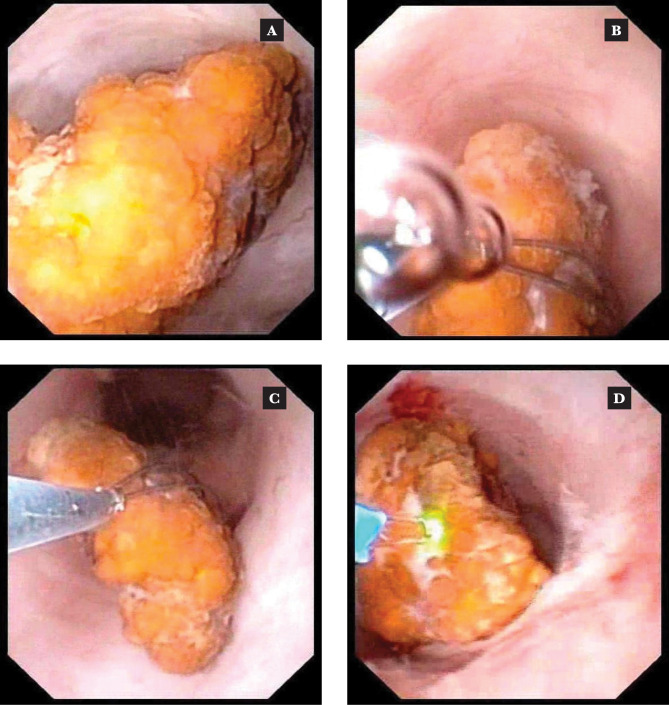
Intraoperative images of stone relocation. A – stone located in the inferior calyx; B, C – relocating the stone to the upper calyx; D – laser lithotripsy of the upper calyx.

Stone replacement is one of the most common techniques several authors have described in managing lower renal pole stones. The success rate of fURS in treating lower pole stones has increased thanks to a procedure that involves moving the stone to a more accessible calyx using tipless Nitinol baskets before laser lithotripsy (Figure 1 A–D).

Compared to the thinnest laser fiber, baskets result in a little loss of irrigation flow and endoscope active deflection, enabling effective access to the lower pole with improved visibility [45-48]. Gokce et al. [49] compared SWL with fURS in 67 patients, emphasizing the advantage of calculus repositioning from the lower calyx level to increase the success of the intervention, resulting in a stone-free rate of 73.9% in fURS.

Flexible ureteroscopes typically consist of a working channel, a deflection mechanism, and an optical system that utilizes fiber-optic images and light bundles. The most important feature of current ureteroscopes is the deflection mechanism, which theoretically allows the visualization of the entire pyelocaliceal system. Over time, the deflection systems of ureteroscopes have undergone significant advancements. Recently, deflection angles were increased to 275 degrees, allowing ureteroscopic tips to access even the farthest point of lower minor calyces. Table 1 provides a comparison of the commonly used ureteroscopes [50,51].

**Table 1:** The main characteristics of various flexible ureteroscopes in current use [50,51].

Characteristics	Pusen	Boston Scientific	Olympus		Karl Storz	Richard Wolf
	PU3033A	LithoVue	URF-P5,6	URF-V2	Flex-X2	Viper
Use	Single-use	Single-use	Reusable	Reusable	Reusable	Reusable
Tip diameter (Fr)	7.5	7.7	4.9	8.4	7.5	6
Shaft diameter (Fr)	7.5	9.5	7.95	8.5	7.5	8.8
Working length (mm)	650	680	670	670	670	680
Channel size (Fr)	3.6	3.6	3.6	3.6	3.6	3.6
Deflection angle	2700/2700	2700/2700	1800/2750	2750	2700	2700

**Figure 2:**
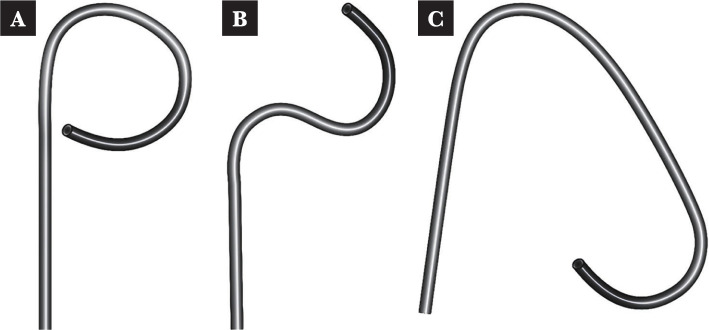
Graphic representation of active and passive deflection in different situations.

**Figure 3:**
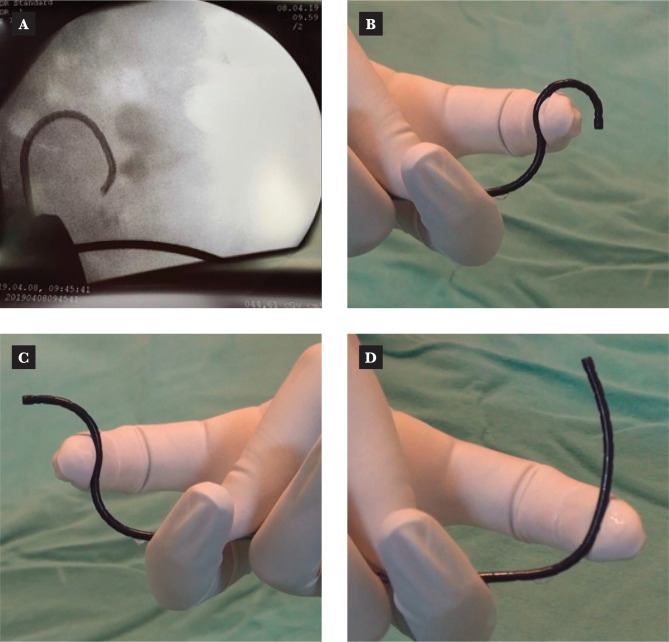
Intraoperative images of active and passive deflection during fURS. A – intraoperative fluoroscopic image; B, C, D – ex. vivo representation of passive deflection).

However, some authors suggest that even with this wide deflection, in many cases, it is difficult to access the lower renal pole or kidneys with anatomical anomalies, so other "tips and tricks" methods must be used to advance anywhere in the pyelocaliceal system. In some situations, active deflection is not enough, and passive deflection is defined as bending the tip of the ureteroscope by supporting it against the calyx (most frequently) or another intrarenal structure to "passively" orient the ureteroscope optics in the opposite direction [52]. Although the concept of using a ureteroscope with two deflections, one active and one passive, was described in 1992 [53] and was very popular at the time, the advancement of technology, the digitalization of ureteroscopes, and their decreasing size led to a relative decrease in the popularity of this approach [54]. However, despite these technological advances, there are still situations in which certain areas of interest cannot be accessed during renoureteroscopies, and therefore, these "tips and tricks" remain valuable tools in the hands of expert urologists (Figures 2 A-C and 3 A-D).

Passive deflection allows for the extension of the deflected section, providing complete inspection and treatment of the intrarenal collecting system. The more flexible section of the ureteroscope, placed just proximal to the site of active deflection, enables secondary passive deflection, facilitating the bending of the angled tip of the ureteroscope off the superior border of the renal pelvis or the neck of a middle-pole calyx when there is a baggy extrarenal pelvis. This effectively extends the ureteroscope's tip, enabling better visualization of the lower pole calyces. The use of passive deflection in combination with active deflection enables a larger proportion of the renal cavities to be visualized with relative ease [55,56]. New-generation ureteroscopes with small calibers, such as Pusen 3022A or 3033A, have emerged as a potential solution to the challenge of accessing difficult-to-reach areas of the pyelocaliceal system. These ureteroscopes offer improved maneuverability, deflection, and limb fatigue performance and are at least competitive with regular f-URS in these aspects. The intrarenal visualization provided by these small-caliber ureteroscopes is comparable to that of the ureteroscopes commonly used in clinical practice. The significant advantage of these devices is their disposability, as they can be easily replaced at any time with minimal costs, making them a cost-effective option for tackling complex cases that would otherwise require the use of expensive, reusable tools [57,58].

**Figure 4:**
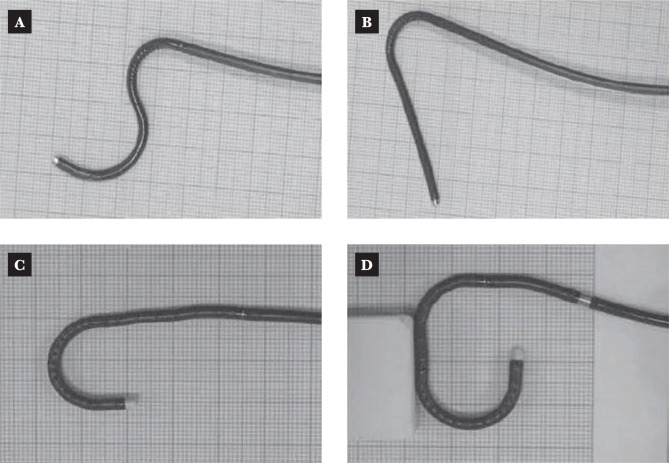
Examples of in-vitro passive deflection of flexible ureteroscope.

**Figure 5:**
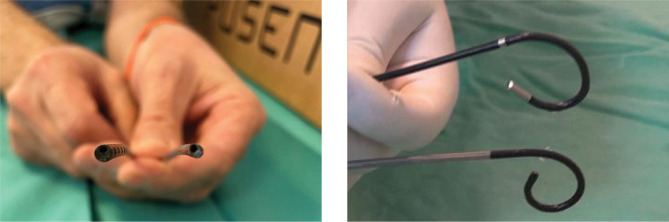
Differences between the 9.5 Fr Pusen (Zhuhai Pusen Medical Technology™) flexible ureteroscope and the new 7.5 Fr ultra-thin flexible ureteroscope.

Different single-use fURS from the authors' collection are represented in Figure 4 A-D and Figure 5.

## DISCUSSION

The introduction of flexible ureteroscopy was one of the technological advancements that improved the surgical treatment of renal stones throughout the last three decades (fURS). This tool has undergone numerous improvements, such as auxiliary equipment like graspers and baskets, the lithotripsy technique with Holmium: YAG laser, or newer technology such as Thulium laser, which have led to the expansion of its indications, to include the diagnostic and therapeutic management of upper urinary tract pathologies like urolithiasis and urothelial tumors [59-64].

Despite the new technological advances, the literature still describes multiple situations where accessing the pelvicalyceal system with the ureteroscope can be challenging. These difficulties may stem from a range of factors, including renal malformations, unique local anatomy, or the technical characteristics of the endoscope. Multiple studies have evaluated the safety profile and success rates of the procedure in abnormal kidneys, and the findings consistently demonstrate low complication rates. However, the stone-free rate did not exceed 90% in nearly all cases [32,65-67]. A 2017 study by Ergin et al. [68] assessed success rates (stone-free) in 101 patients with renal lithiasis and associated renal anatomical anomalies. The study found that patients with horseshoe kidneys had success rates of 72.2% for fURS and 90% for PCNL, while those with ectopic kidneys had success rates of 83.6% for fURS and 100% for laparoscopic pyelolithotomy. Additionally, patients with renal rotation abnormalities had stone-free rates of 75% for horseshoe kidneys and 83.3% for PCNL. These results suggest that although flexible ureteroscopy has lower complication rates than other kidney stone treatment methods, its success may be limited in special cases due to technical difficulty. Even in normal, conforming kidneys, there are certain situations where access of the ureteroscope is made difficult by the unfavorable local anatomy. The literature describes the most frequent situation when the calculus is associated with the lower calyx. According to a 2017 study [69], digital fURSs had limited end-tip deflection compared to fiberoptic fURSs and were less successful at reaching the inferior calyx's acute angle. As a result, it may be preferable to utilize a fiberoptic fURS while attempting to approach a challenging inferior calyx. Renoscopy for lower calyx calculus may involve relocating the calculus to another calyx using a nitinol basket [70] so that the angulation of the ureteroscope during lithotripsy is not very steep. This approach can improve the visualization of the calculus and protect the endoscope from unintended damage.

Another predictive factor for the success of ureteroscopy is the local intrarenal anatomy. Multiple studies have evaluated IPA, IL, and CPH [71,72]. A study by Tomasz Ozimek et al. (2018) [73] evaluated 381 fURS in terms of IPA, ureteroscope damage, and complication rates. It concluded that a steep IPA (<60°) is associated with higher rates of complications and an increased possibility of endoscope malfunctions. However, the latest study on this topic suggests that an IPA of <45.8° is a poor indicator for reaching the lower pole of the kidney during fURS.

When the area of interest cannot be properly visualized, another described technique is “passive deflection”. Only the distal tip of the ureteroscope experiences active deflection, and the deflected segment might not be long enough to reach the lower pole calyx. Due to a weakness in the durometer of the sheath, which is situated close to the point of active deflection, the majority of flexible ureteroscopes feature a more flexible segment of the ureteroscope [74]. This additional passive deflection mechanism is responsible for an additional deflection when the active one is not enough. The point of deflection on the ureteroscope is effectively pushed more proximally, expanding the tip of the ureteroscope by passively bending the tip off the superior border of the renal pelvis. In most patients, the lower pole calyx may be reached with passive deflection. However, there are also particular situations, such as patients with a high degree of hydronephrosis, which can make it difficult to use passive secondary deflection.

## Conclusion

Flexible ureteroscopy has become an essential tool in the arsenal of modern urologists for the treatment of renal lithiasis. Technological advances have made this procedure safe, and efficient, and provided excellent results in the benefit-safety ratio. However, achieving maximal success rates in terms of stone-free status can be challenging, and multiple parameters such as anatomical anomalies or special characteristics of the intrarenal anatomy must be considered before any procedure. Nevertheless, experienced surgeons can increase the success rate using various “tips and tricks” maneuvers tailored to individual cases.
